# Performance of AI Approaches for COVID-19 Diagnosis Using Chest CT Scans: The Impact of Architecture and Dataset

**DOI:** 10.1055/a-2577-3928

**Published:** 2025-04-29

**Authors:** Astha Jaiswal, Philipp Fervers, Fanyang Meng, Huimao Zhang, Dorottya Móré, Athanasios Giannakis, Jasmin Wailzer, Andreas Michael Bucher, David Maintz, Jonathan Kottlors, Rahil Shahzad, Thorsten Persigehl

**Affiliations:** 1 Institute for Diagnostic and Interventional Radiology, Faculty of Medicine and University Hospital Cologne, University of Cologne, Cologne, Germany; 2 117971 Department of Radiology, The First Hospital of Jilin University, Changchun, China; 3 Department of Diagnostic and Interventional Radiology, University Hospital Heidelberg, University of Heidelberg, Heidelberg, Germany; 4 Institute for Diagnostic and Interventional Radiology, Frankfurt University Hospital, Frankfurt, Germany; 5 Philips Healthcare, Innovative Technologies, Aachen, Germany

**Keywords:** Artificial Intelligence, Pandemic Preparedness, CT Scan

## Abstract

**Purpose:**

AI is emerging as a promising tool for diagnosing COVID-19 based on chest CT scans. The aim of this study was the comparison of AI models for COVID-19 diagnosis. Therefore, we: (1) trained three distinct AI models for classifying COVID-19 and non-COVID-19 pneumonia (nCP) using a large, clinically relevant CT dataset, (2) evaluated the models’ performance using an independent test set, and (3) compared the models both algorithmically and experimentally.

**Materials and Methods:**

In this multicenter multi-vendor study, we collected n=1591 chest CT scans of COVID-19 (n=762) and nCP (n=829) patients from China and Germany. In Germany, the data was collected from three RACOON sites. We trained and validated three COVID-19 AI models with different architectures: COVNet based on 2D-CNN, DeCoVnet based on 3D-CNN, and AD3D-MIL based on 3D-CNN with attention module. 991 CT scans were used for training the AI models using 5-fold cross-validation. 600 CT scans from 6 different centers were used for independent testing. The models’ performance was evaluated using accuracy (Acc), sensitivity (Se), and specificity (Sp).

**Results:**

The average validation accuracy of the COVNet, DeCoVnet, and AD3D-MIL models over the 5 folds was 80.9%, 82.0%, and 84.3%, respectively. On the independent test set with n=600 CT scans, COVNet yielded Acc=76.6%, Se=67.8%, Sp=85.7%; DeCoVnet provided Acc=75.1%, Se=61.2%, Sp=89.7%; and AD3D-MIL achieved Acc=73.9%, Se=57.7%, Sp=90.8%.

**Conclusion:**

The classification performance of the evaluated AI models is highly dependent on the training data rather than the architecture itself. Our results demonstrate a high specificity and moderate sensitivity. The AI classification models should not be used unsupervised but could potentially assist radiologists in COVID-19 and nCP identification.

**Key Points:**

- This study compares AI approaches for diagnosing COVID-19 in chest CT scans, which is essential for further optimizing the delivery of healthcare and for pandemic preparedness.
- Our experiments using a multicenter, multi-vendor, diverse dataset show that the training data is the key factor in determining the diagnostic performance.
- The AI models should not be used unsupervised but as a tool to assist radiologists.

**Citation Format:**

- Jaiswal A, Fervers P, Meng F et al. Performance of AI Approaches for COVID-19 Diagnosis Using Chest CT Scans: The Impact of Architecture and Dataset. Rofo 2026; 198: 185–198

## Zusammenfassung

**Ziel:**

Aktuell existieren verschiedenste Künstliche Intelligenz (KI)-Modelle zur Detektion und Klassifikation von Pneumonien in Thorax-CTs, aber unabhängige Vergleiche fehlen meist. In dieser Studie haben wir (1) drei verschiedene KI-Modelle zur Klassifizierung von COVID-19- und Nicht-COVID-19-Pneumonien (nCP) anhand eines klinisch relevanten CT-Datensatzes trainiert, (2) die Leistung der Modelle anhand eines unabhängigen Testsatzes bewertet und (3) die Modelle sowohl algorithmisch als auch experimentell verglichen.

**Materialien und Methoden:**

In dieser multizentrischen, retrospektiven Studie haben wir insgesamt 1591 Thorax-CTs von COVID-19- (n=762) und nCP (n=829)-Patienten aus China und Deutschland zusammengestellt; in Deutschland wurden die CT-Daten von 3 RACOON-Standorten eingeschlossen. Es wurden 3 open-source KI-Modelle mit unterschiedlichen Architekturen trainiert und validiert: COVNet basierend auf 2D-CNN, DeCoVnet basierend auf 3D-CNN, und AD3D-MIL basierend auf 3D-CNN mit Attention-Modul. Die Performance der Modelle wurde anhand von Genauigkeit (Acc), Sensitivität (Se) und Spezifität (Sp) bewertet.

**Ergebnisse:**

Die durchschnittliche Validierungsgenauigkeit der Modelle COVNet, DeCoVnet und AD3D-MIL über die 5-Fach-Validierung im Training mit n=991 CTs betrug 80,9%, 82,0% bzw. 84,3%. Auf dem unabhängigen Testsatz mit n=600 CTs lieferte COVNet: Acc=76,6%, Se=67,8%, Sp=85,7%; DeCoVnet: Acc=75,1%, Se=61,2%, Sp=89,7%; und AD3D-MIL: Acc=73,9%, Se=57,7%, Sp=90,8%.

**Schlussfolgerung:**

Die Klassifizierungsleistung der evaluierten KI-Modelle hängt in hohem Maße von den Trainingsdaten und weniger von der Architektur selbst ab. Unsere Ergebnisse zeigen eine hohe Spezifität und eine moderate Sensitivität bei der Differenzierung von COVID-19- und Nicht-COVID-19-Pneumonien. Die KI-Klassifikationsmodelle sollten aber nicht unkritisch verwendet werden, könnten aber Radiologen unterstützen.

**Kernaussagen:**

- Vorliegende Studie vergleicht KI-Ansätze zur bildbasierten Diagnose von COVID-19 in Thorax-CTs, was relevant für die weitere Optimierung der Gesundheitsversorgung und die Vorbereitung auf etwaig kommende Pandemien ist.
- Unser multizentrischer, herstellerübergreifender Datensatz zeigt, dass die Trainingsdaten der entscheidende Faktor für die diagnostische Leistungsfähigkeit sind.
- KI-Modelle sollten nicht autonom eingesetzt werden, sondern als unterstützendes Werkzeug in die radiologische Befundung integriert werden, um die diagnostische Entscheidungsfindung zu ergänzen – nicht zu ersetzen.

## Abbreviations

AI: Artificial intelligence
CapsNet: Capsule network
CNN: Convolutional neural network
COVID-19: Coronavirus disease 2019
CT: Computed tomography
HU: Hounsfield unit
MLP: Multi-layer perceptron
nCP: Non-COVID-19 pneumonia
ResNet: Residual network
RT-PCR: Reverse transcription polymerase chain reaction test
CI: Confidence interval
ROC: Receiver operating characteristic curve
AUC: Area under the curve

## Introduction

The coronavirus disease 2019 (COVID-19) pandemic is a poignant reminder of how rapidly a global health crisis can emerge and pose a significant challenge to the world's healthcare systems. The potential for other pathogens to cause future pandemics, with the lungs as a primary target, underscores the critical need for comprehensive pandemic preparedness measures. As with COVID-19, fast detection and prompt patient isolation will be crucial for curbing the spread of future pandemics. Chest computed tomography (CT) is a method of choice for COVID-19 and other viral and bacterial pneumonias 1 . Due to a lack of alternative diagnostic methods in the early pandemic, chest CT was frequently performed to diagnose the disease 2 . The SARS-CoV-2 reverse polymerase chain reaction (RT-PCR) and antibody test has since become widely accessible and is considered the most reliable method for diagnosing COVID-19 3 . Nevertheless, chest CT still has a potential role in the diagnosis of COVID-19 pneumonia and for determining disease stage 1 . The Fleischner Society recommends chest CT as a diagnostic tool, if RT-PCR resources are limited and could delay isolation or crucial treatment 4 . Furthermore, a patient with a suspected false-negative RT-PCR test and at least moderate clinical features qualifies for a chest CT scan 4 . Although the diagnosis of COVID-19 is currently the domain of laboratory testing, chest CT is frequently performed to provide detailed information about the severity and extent of lung involvement 5 .

With the aim of supporting radiologists, numerous AI approaches have been developed for the automatic detection of COVID-19 based on CT scans. These algorithms are often based on convolution neural networks (CNN) with two dimensions ( *2D* ) 6 7 8 9 10 11 12 13 or three dimensions ( *3D* ) 14 15 16 17 18 . *2D* approaches learn features from individual slices in a volumetric CT scan 6 7 8 9 10 11 12 13 . Slice-level results are often aggregated to obtain patient-level predictions. *3D approaches* , on the other hand, utilize 3D volume for feature extraction and directly generate patient-level predictions 14 15 16 17 18 . Different approaches require *patient-level* 9 10 14 15 18 , *slice-level* 8 , or *pixel-level* 19 labels for training. Along with conventional CNNs, *machine-driven design* 20 21 has also been explored. *Hybrid strategy* , which employs traditional machine learning along with deep learning 22 has also been proposed. A summary of various algorithms proposed in the literature is shown in Table 1 .

**Table:** **Table 1** Summary of AI algorithms proposed in the literature for COVID-19 diagnosis.

COVID-19 diagnosis algorithm	2D CNN	3D CNN	Caps-Net	Pixel-level label	Slice-level label	Patient-level label	Machine-driven design	Hybrid
Xiong et al. (2020) 6 , Rahimzadeh et al. (2021) 8 , Wang et al. (2021) 12 , Wang et al. (2021) 13	X				X			
Song et al. (2021) 7 , Jin et al. (2020) 9 , Li et al. (2020) 10	X					X		
Qian et al. (2020) 11	X				X	X		
Wang et al. (2020) 14 , Han et al. (2020) 15 , Lee et al. (2021) 16 , Javaheri et al. (2021) 17 , Wang et al. (2020) 18		X				X		
Zhang et al. (2020) 19		X		X				
Wu et al. (2021) 23	X			X		X		
Amyar et al. (2020) 24 , Wang et al. (2021) 25 , Gao et al. (2021) 26	X			X				
Afshar et al. (2022) 27			X	X				
Qi et al. (2022) 28			X		X			
Gunraj et al. (2020) 20 , Gunraj et al. (2022) 21	X						X	
Qi et al. (2021) 22 , Mei et al. (2020) 29	X					X		X
Hou et al. (2021) 30	X	X			X	X		

Many algorithms proposed in the literature lack an external validation dataset (e.g., 8 10 11 ), which is important to assess the generalization of the AI models. An independent comparison of multiple approaches based on a common dataset can play a guiding role for both radiologists and AI developers. To address these issues, in this study, we collected a large and diverse set of CT scans from China and Germany. Chest CT data from three RACOON sites (Cologne, Frankfurt, Heidelberg) have been utilized in this study. RACOON is a nationwide RAdiological COOperative Network of 36 university hospitals in Germany. RACOON is supported by the National University Medicine Network (NUM) founded by the German Federal Ministry of Education and Research (BMBF). The unique RACOON infrastructure supports large-scale AI studies and could play a key role in Germany’s pandemic preparedness program.

Using datasets from China and Germany in this study, we aimed to assess and compare the performance of three distinct AI approaches (based on 2D and 3D CNNs) for distinguishing between COVID-19 pneumonia and non-COVID-19 pneumonia (nCP). Our overall goal was the assessment of three publically available AI tools for COVID-19 diagnosis and to determine whether these AI tools could potentially be used to support radiologists in clinical decision making.

## Materials and methods

### Dataset

**Fig. 1:**
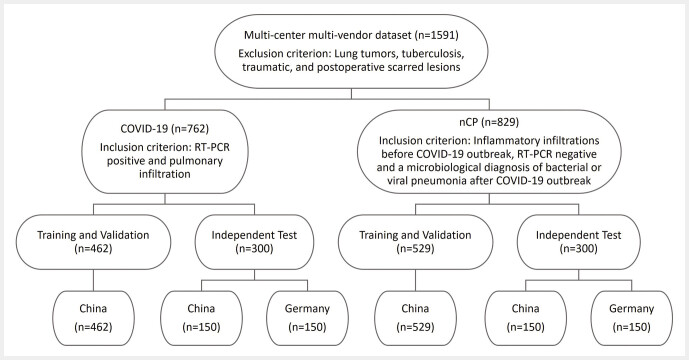
Distribution of our multicenter, multi-vendor dataset including classes, training and test sets, and countries. Exclusion criterion as well as inclusion criterion per class (COVID-19, nCP) are also shown.

For this retrospective IRB-approved study, we collected a multicenter, multi-vendor chest CT dataset consisting of n=1591 chest CT scans of COVID-19 (n=762) and nCP (n=829) patients from China and Germany ( Fig. 1 ). For the COVID-19 class, the inclusion criteria were pulmonary infiltration, and a positive RT-PCR test within 48 h before the CT examination. For the nCP class, the inclusion criteria were: (1) inflammatory infiltrations on CT scans before the outbreak of COVID-19 (from February 2016 to December 2019), (2) an additional negative RT-PCR test after the outbreak of COVID-19 (January 2020). The exclusion criteria were imaging features consistent with lung tumors, tuberculosis, and traumatic and postoperative scarred lesions.

Our dataset is balanced across the two disease classes. The COVID-19 class includes cases with different disease stages (early: 0–3 days, progressive: 4–7 days, peak: 8–14 days, and absorption: ≥15 days) assessed based on CT morphology and the gap between CT scanning and symptom onset 31 . Based on laboratory etiological confirmation, the nCP class includes pneumonia caused by viral and bacterial pathogens. The number of scans and distribution over the two classes and the subcategories for training and test dataset are presented in Table 2 . Detailed patient demographics across different centers are given in Table 3 . The entire Chinese dataset, which contains only full-dose CT scans, was imaged using seven different CT device manufacturers. The dataset from Germany, which contains 53.7% low-dose CT scans and 46.3% full-dose CT scans, was imaged using two CT device manufacturers ( Table 3 ). Further information about vendors, protocols, etc. can be found in the recent article 32 .

**Table:** **Table 2** Imaging stages of COVID-19 and etiological classification of nCP. Number of samples in training and test datasets as well as the stages/etiology distribution are presented.

Disease	Stages/etiology	Training dataset (n)	Test dataset (n)	Distribution (%)
COVID-19	Early	53	101	20.2
	Progressive	292	82	49.1
	Peak	48	68	15.2
	Absorption	69	49	15.5
nCP	Bacterial	197	150	41.8
	Viral	135	150	34.4
	N/A	197	0	23.8
Note: N/A (not available) refers to the nCP patients, who did not test for etiology, or the cases in which the results of the etiological tests were negative.				

**Table:** **Table 3** Demographic values, scanner companies, and slice thickness of the entire dataset according to country, center, and class. Neusoft Healthcare (NH), Minfound Healthcare (MH), and United imaging Healthcare (UIH).

Country	Center	COVID-19			nCP			CT vendors (n)						
			Sex	Age		Sex	Age							
		Scans (n)	Male, female (n)	Mean ± Std, (range)	Scans (n)	Male, female (n)	Mean ± Std, (range)	GE	Philips	Siemens	TOSHIBA	NH	MH	UIH
China	Jilin	69	35, 26	41.8 ± 12.9 (16, 62)	299	167, 130	57.1 ± 16.0 (18, 95)	31	142	86	5	99	5	
	Wuhan	446	196, 249	57.8 ± 15.1 (15, 98)	292	148, 143	57.4 ± 18.1 (4, 89)	557	3	178				
	Ningbo	97	36, 61	50.4 ± 14.3 (17, 86)	88	69, 19	69.0 ± 13.6 (32, 90)		2	152			21	10
Germany	Cologne	50	26, 24	59.7 ± 14.1 (29, 88)	50	28, 22	59.6 ± 18.9 (18, 89)		100					
	Frankfurt	50	42, 8	58.5 ± 13.9 (36, 85)	50	32, 18	59.9 ± 13.3 (21, 90)		9	91				
	Heidelberg	50	34, 16	56.9 ± 15.6 (20, 85)	50	34, 16	58.9 ± 15.4 (19, 86)		76	24				
Total		762	369, 384	54.2 ± 14.3 (15, 98)	829	478, 348	60.3 ± 15.9 (4, 95)	588	332	531	5	99	26	10
Note: COVID-19 – coronavirus disease 2019; nCP – non-COVID-19 pneumonia, number; ages are reported as means ± standard deviation														

We used n=991 CT scans (n=462 COVID-19 and n=529 nCP CT scans) from three different centers in China to train the AI models using a five-fold cross-validation approach. In each fold, non-overlapping 80% of the CT scans were used for training and 20% of the scans were used for validation. CT scans from all six centers from China (n=300; centers: Jilin, Wuhan, Ningbo) and Germany (n=300, external dataset; centers: Cologne, Frankfurt, Heidelberg) were used for independent testing to get an indication of the generalization of the trained models. The test set contained a total of n=600 CT scans with balanced COVID-19 and nCP classes, which the algorithms did not see during training or validation. Therefore, this test set is called the independent test set. The internal test set came from the same sites as the training dataset. The external test set came from different sites. An in-detail description of the training and validation process was recently published 32 .

### Automatic COVID-19 diagnosis

**Fig. 2:**
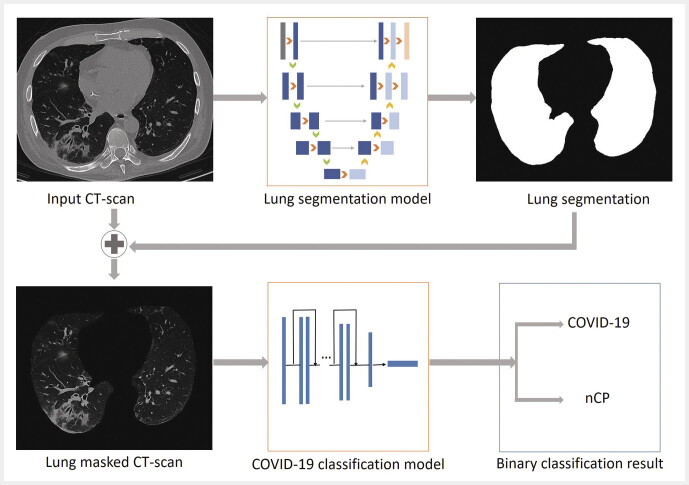
Overview of the three evaluated COVID-19 diagnostic algorithms. The lung segmentation model accepts a CT scan as input and generates lung masks, which are concatenated with the original images. Lung masked CT scans are used by the classification model to detect COVID-19. Note: the implementation details may vary for each of the diagnostic methods.

For this study, we selected AI algorithms based on criterion such as: (1) different network architectures and training strategies, (2) availability of code and documentation, and (3) ability to train with only patient-level labels. For more details about our literature search and model selection, see **supplement 1** . We trained and validated the selected models for diagnosing COVID-19 using chest CT scans: COVNet 10 based on 2D-CNN, DeCoVnet 14 based on 3D-CNN, and AD3D-MIL 15 based on 3D-CNN with an attention module. All three approaches consisted of two steps ( Fig. 2 ). The first step segments the lung area to avoid the effect of irrelevant regions. The second step classifies the lung-masked CT scans as COVID-19 or nCP.

### Lung segmentation

For lung segmentation, we utilized three different models: Seg-Net 33 , U-Net 9 34 , and U-Net(R-231) 34 35 . The models were trained on different datasets and employ slightly different preprocessing. The Seg-Net 33 model was trained and tested using 44,500 CT slices. Preprocessing involved resampling (1×1 mm), rescaling (512×512), windowing [-1000, 500 HU], and normalization (0–1). The 2D U-Net 9 34 model was trained and tested using 16,223 CT slices. Preprocessing included resampling (1×1mm), windowing [-1200, 700 HU], and normalization (0–1). The U-Net(R-231) 35 model is based on U-Net 34 with batch normalization and was trained on a diverse dataset of 62,224 CT slices. Preprocessing included body cropping, rescaling (256×256), windowing [-1024, 600 HU], and normalization (0–1).

### COVID-19 classification

The literature review (see **supplement 1** ) yielded the following three algorithms for assessment in this study:

#### 1. COVID-19 detection neural network (COVNet)

**Fig. 3:**
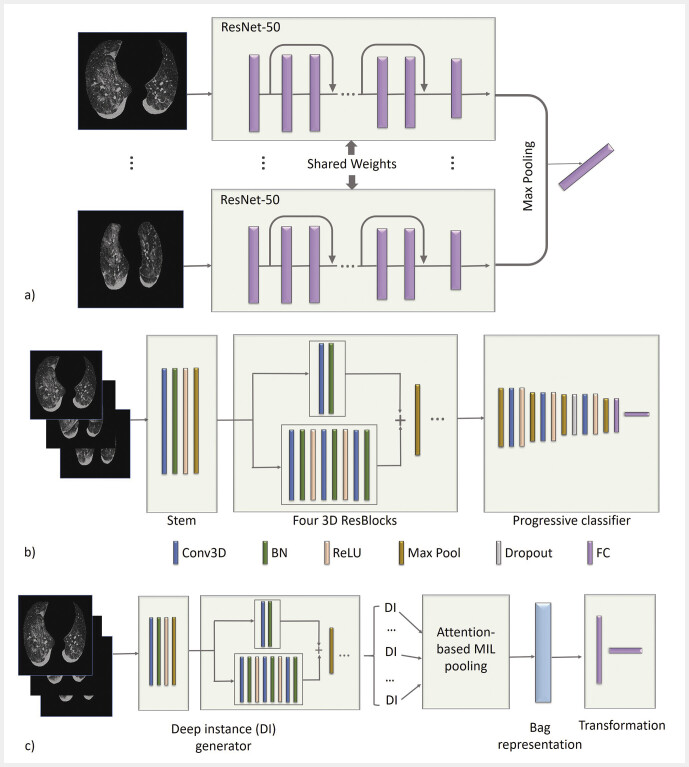
Schematic overview of ( **a** ) COVNet, ( **b** ) DeCoVnet, and ( **c** ) AD3D-MIL. **a** The ResNet-50 backbone of COVNet generates features from each slice, which are flattened, and combined to generate a global feature vector using max pooling. **b** DeCoVnet consists of stem, ResBlocks, and progressive classifier stages. Different colors represent different operations. It includes 3D convolution layers (Conv3D), batch normalization (BN), max pooling (Max Pool), dropout, and fully connected layers (FC). **c** AD3D-MIL utilizes ResBlocks from DeCoVnet for deep instance generation. Transformer function is implemented using two fully connected layers.

COVNet is based on ResNet-50 36 , which is a 50-layer residual network. COVNet takes a lung-masked CT scan as input and provides a patient-level prediction as output. As shown in Fig. 3 (a), ResNet-50 captures slice-level features using 2D convolutions (2D CNN). CT-level features are obtained by max. pooling. Preprocessing steps include resampling (224×224 in-plane), down sampling (by a factor of 5 in the Z-direction), intensity clipping (-1250, 250 HU), and normalization (0, 1). During training, data augmentation was used by applying random rotation, flipping, and adding Gaussian noise to the input data. The weights of ResNet-50 were initialized using weights optimized on the ImageNet database 37 .

#### 2. 3D deep convolutional neural network to detect COVID-19 (DeCoVnet)

In contrast to COVNet, DeCoVnet is based on 3D ResNet which performs 3D convolutions (3D CNN) to learn features. As shown in Fig. 3 (b), DeCoVnet consists of the network stem, residual blocks (ResBlocks), and progressive classifier stages. The four ResBlocks include shortcut connections and pass 3D feature maps. The classifier progressively extracts important features using 3D max pooling and directly yields CT-level class probabilities. Input to DeCoVnet is a lung-masked CT scan. Preprocessing included resampling (224×336), intensity clipping (-1200, 600 HU), and normalization (0, 1). During training, data augmentation was performed that included random affine transformations and color jittering. Weights of the model were initialized using the Kaiming initialization method 38 .

#### 3. Attention-based deep 3D multiple instance learning (AD3D-MIL)

AD3D-MIL addresses the problem of detecting COVID-19 as multiple instance learning (MIL), where instances are automatically generated using convolution layers of DeCoVnet 14 . Once instances are obtained, attention-based pooling is applied to concentrate on important instances by weighting them. Next, a two-layer fully connected neural network provides final class probabilities. Compared to the above two methods, AD3D-MIL is based on 3D ResNet with attention pooling ( Fig. 3 (c)). AD3D-MIL takes lung-masked CT scans as input. Preprocessing was performed by using resampling (256×256), intensity clipping (-1024, 600 HU), and normalization (0, 1). Data augmentation included color jittering and random affine transformation. For training, the model was initialized using random weights following Kaiming initialization 38 .

### Training

For diagnosing COVID-19, we trained, validated, and tested the three models (COVNet, DeCoVnet, and AD3D-MIL) using exactly the same set of images. The models were trained using a five-fold cross-validation approach. Based on the highest validation accuracy (Acc), the best model was selected from each fold for inference. Pretrained models were used for lung segmentation. We used the Seg-Net 33 obtained lung masks for the COVNet model, U-Net 9 lung masks for DeCoVnet, and U-Net(R-231) 35 lung masks for AD3D-MIL in accordance with the original 14 15 or previous publications 32 . Hyperparameters are presented in **supplementary Table 2** . During inference on the independent test set, the predictions from the five best models were ensembled using majority voting.

### Statistics

Statistical analysis was performed in Python using SciPy (Stats) 39 and Scikit-learn (Metrics, Calibration) 40 packages and in R software using pROC 41 package. Figures were plotted using the Matplotlib (Pyplot) package 42 . Statistical hypothesis testing of the non-parametric dichotomous performance data was calculated from pairwise 2×2 contingency tables using McNemar’s test. A bootstrapping approach was applied to calculate the confidence interval. DeLong’s test was used to compare AUCs. Statistical significance was defined as *p* < .05.

## Results

### Lung segmentation

**Fig. 4:**
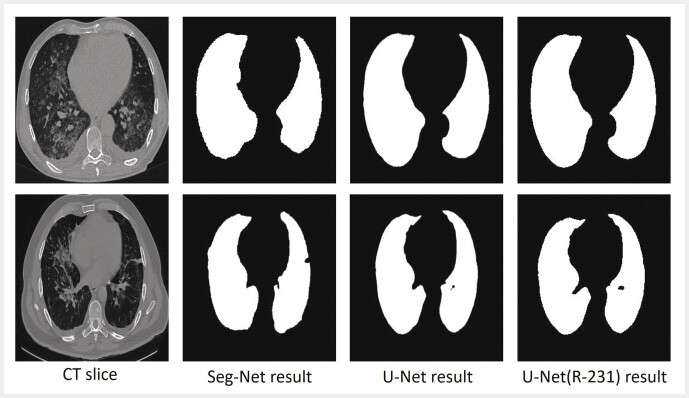
Lung masks obtained by using different lung segmentation models. A slice from an nCP (viral pneumonia) CT scan (top row) and a slice from a COVID-19 CT scan (bottom row) are shown.

For lung segmentation, we used three different CNN models: Seg-Net 33 , U-Net 9 34 , and U-Net(R-231) 34 35 , trained on different datasets. Upon visual analysis, we found that the lung masks produced by all three models were of sufficient quality. Fig. 4 shows exemplary slices from nCP and COVID-19 CT scans along with the lung masks obtained by using the three lung segmentation models.

### COVID-19 classification

**Fig. 5:**
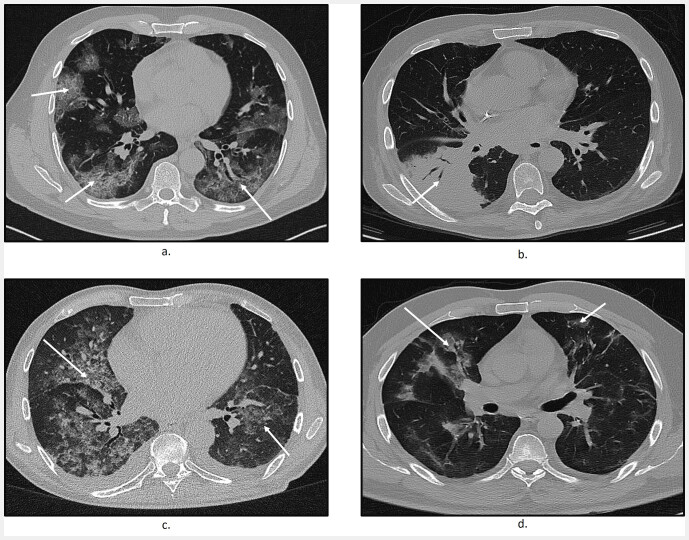
Slices from CT scans of nCP (viral pneumonia, bacterial pneumonia) and COVID-19 patients as well as predictions from the three diagnostic models. **a** Exemplary axial slice of a CT scan of a COVID-19 patient displaying bilobular peripheral GGO and consolidations (see arrows). The disease stage is progressive. COVNet, DeCoVnet, and AD3D-MIL predicted the scan as COVID-19-positive. **b** Exemplary axial slice of a CT scan of a bacterial pneumonia patient with unilobular consolidation (see arrows). All three diagnostic models predicted the scan as nCP-positive. **c** Exemplary axial slice of a CT scan of a viral pneumonia patient with bilobular reticular consolidations and central GGOs (see arrows). The disease stage was categorized as extensive. COVNet and DeCoVnet predicted the scan as nCP-positive whereas AD3D-MIL predicted it incorrectly as COVID-19-positive. **d** Exemplary axial slice of a CT scan of a COVID-19 patient in absorption stage with bilobular consolidations and septal thickening (see arrows). All three AI models COVNet, DeCoVnet, and AD3D-MIL predicted the scan incorrectly as nCP-positive.

In Table 4 , we present the performance (validation accuracy) of the best models from the five folds on the corresponding validation data. CV1 to CV5 represent cross-validation folds 1 to 5. The mean accuracy obtained by the COVNet, DeCoVnet, and AD3D-MIL models over the 5 folds are 80.9%, 82.0%, and 84.3%, respectively. In Fig. 5 , we show slices from CT scans of nCP (viral pneumonia, bacterial pneumonia) patients and COVID-19 patients as well as the corresponding patient-level predictions provided by the three diagnostic models. These scans depict different patterns including unilobular or bilobular infiltrations, and different disease stages or etiologies. As can be seen from these examples, all three COVID-19 diagnostic models yielded similar predictions.

**Table:** **Table 4** Performance of the three COVID-19 diagnostic AI models with respect to the validation dataset, obtained during different cross-validation folds (CV1 to CV5). In addition, performance of the models with respect to the independent test set (600 scans from China and Germany), including accuracy, sensitivity, and specificity along with the 95% confidence interval (CI) and Brier score are presented. ACC-DE, AUC-DE and ACC-CH, AUC-CH are accuracies and AUCs for the test data from Germany and China, respectively.

AI model for COVID-19 detection	Performance (Acc (%)) with respect to validation set (20% of the training set)		Performance with respect to independent test set								
			Test set Germany (300 CT scans)		Test set China (300 CT scans)		Test set (Germany and China, 600 CT scans)				
	CV1, CV2, CV3, CV4, CV5	Mean±Std	Acc-DE (%) [CI]	AUC-DE [CI]	Acc-CH (%) [CI]	AUC-CH [CI]	Acc (%) [CI]	Se (%) [CI]	Sp (%) [CI]	AUC [CI]	Brier score [CI]
COVNet	78.5, 84.8, 80.6, 78.5, 81.9	80.9±2.4	75.7 [70.7, 80.4]	0.84 [0.79, 0.89]	77.5 [72.8, 82.5]	0.87 [0.83, 0.91]	76.6 [73.2, 80.0]	67.8 [62.5, 72.9]	85.7 [81.5, 89.8]	0.86 [0.83, 0.89]	0.16 [0.14, 0.18]
DeCoVnet	81.7, 80.1, 82.2, 82.2, 83.9	82.0 ±1.2	73.9 [68.6, 78.6]	0.84 [0.79, 0.88]	76.4 [71.4, 81.4]	0.88 [0.84, 0.92]	75.1 [72.0, 78.8]	61.2 [55.4, 66.6]	89.7 [85.9, 93.3]	0.86 [0.83, 0.89]	0.18 [0.15, 0.2]
AD3D-MIL	83.8, 88.5, 82.7, 81.2, 85.5	84.3 ±2.5	68.2 [62.9, 73.9]	0.81 [0.76, 0.86]	79.6 [74.6, 84.3]	0.88 [0.84, 0.92]	73.9 [70.5, 77.9]	57.7 [51.9, 63.1]	90.8 [87.5, 93.9]	0.84 [0.81, 0.87]	0.20 [0.18, 0.23]

**Fig. 6:**
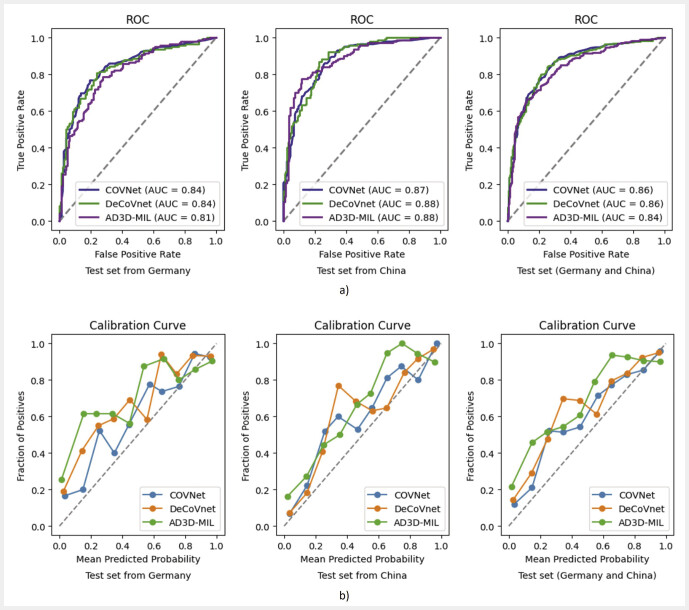
ROC **a** and calibration curves **b** for COVNet, DeCoVnet, and AD3D-MIL for the test set from Germany, China, and both.

We quantified the diagnostic performance using accuracy (Acc), sensitivity (Se), and specificity (Sp). Table 4 presents the performance and the Brier score for the three AI models on the independent test set. COVNet yielded Acc=76.6%, Se=67.8%, Sp=85.7%; DeCoVnet provided Acc=75.1%, Se=61.2%, Sp=89.7%; and AD3D-MIL resulted in Acc=73.9%, Se=57.7%, Sp=90.8%. Each model yielded a moderate sensitivity and a relatively high specificity. The three models achieved similar performance with respect to the independent test set. The difference between the models’ performance was not statistically significant (COVNet vs. DeCoVnet: *p* =.49; COVNet vs. AD3D-MIL: *p* =.20; DeCoVnet vs. AD3D-MIL: *p* =.56). ROC curves, AUCs, and calibration curves for the three models for the test set (including scans from Germany and China) are presented in Fig. 6 . Comparable AUCs (COVNet: 0.86; DeCoVnet: 0.86; AD3D-MIL: 0.84) and Brier scores (COVNet: 0.16; DeCoVnet: 0.18; AD3D-MIL: 0.20) indicate that the models have comparable discrimination performance and levels of calibration.

In addition to the complete test set, performance (accuracy, AUC, ROC and calibration curves) with respect to the test data from Germany (external) and China (internal) is also shown in Table 4 and Fig. 6 . **Supplementary Table 3** presents the DeLong test for comparing AUCs. The AUCs of the three models are not significantly different for any of the internal and external test sets. AD3D-MIL performs better on the internal test set compared to the external test set. AUCs for COVNet and DeCoVnet do not differ significantly between internal and external test sets.

For a comprehensive comparison, we show datasets and classification results from the original publications of COVNet 10 , DeCoVnet 14 , and AD3D-MIL 15 in Table 5 . Additionally, the results obtained from other studies (Hou et al. (2021) 30 , Qian et al. (2020) 11 , Wang et al. (2021) 12 , Wang et al. (2021) 13 ) that compared their approaches with COVNet and DeCoVnet are also shown.

**Table:** **Table 5** Dataset and classification results presented in the original publications of COVNet, DeCoVnet, and AD3D-MIL as well as in other studies comparing their approaches with COVNet and DeCoVnet.

Published study	Dataset	Classes	Perf.	COVNet	DeCoVnet	Theirs
COVNet Li et al. (2020) 10	4352 CT scans, 3322 patients, six centers	COVID-19, CAP, non-pneumonia	AUC	> 0.95	–	–
DeCoVnet Wang et al. (2020) 14	630 CT scans, 540 patients, one center	COVID-19, healthy	Acc (%)	–	90.1	–
AD3D-MIL Han et al. (2020) 15	460 CT scans, 309 patients, multi-center	COVID-19, common pneumonia + no pneumonia	Acc (%)	–	96.1	97.9
Hou et al. (2021) 30	801 scans, 707 patients, inhouse*	COVID-19, H1N1, CAP	Acc (%)	68.2	84.9	90.5
Qian et al. (2020) 11	734 patients, inhouse	COVID-19, H1N1, CAP, healthy	Acc (%)	68.8	93.8	95.2
Wang et al. (2021) 12	1164 CT scans, local hospitals	COVID-19, CAP, SPT, healthy	Se (%)	89.8	91.1	95.6
Wang et al. (2021) 13	640 images, 284 patients	COVID-19, healthy	Acc (%)	93.8	90.3	97.1
* Hou et al. (2021) 30 included two additional public datasets with 2D slices in their experiment. We only mention their 3D dataset and corresponding classification performance because of the focus of our study.						

## Discussion

Since the beginning of the pandemic, CT imaging has played an important role in the diagnosis, severity assessment, and management of COVID-19 disease. In general, given the potential for AI to improve patient care in radiology by aiding in detection and classification tasks, it is crucial to investigate methods and underlying principles for improving these AI algorithms in order to further optimize the delivery of healthcare. While various classification models have been proposed for diagnosing COVID-19 pneumonia on chest CT, this study fills the gap in the literature by performing an independent comparison of three AI models (COVNet, DeCoVnet, and AD3D-MIL) with different architectures for classifying COVID-19 and nCP, and thereby diagnosing COVID-19. Studies by Garg et al. 43 and Ardakani et al. 44 compared different neural networks. However, their models were trained using individual slices, 3D models were not evaluated, and the datasets were relatively small (collected from single country, number of patients n=210 and n=194, respectively). To the best of our knowledge, this is the first study to perform an independent comparison of these approaches using a multicenter, multi-vendor, multi-country dataset. From a clinical perspective, independent performance and robustness assessments are important to evaluate which AI models can potentially be used to support radiologists in clinical decision making.

The three AI models compared in this study have architectural differences (COVNet: 2D-CNN, DeCoVnet: 3D-CNN, and AD3D-MIL: 3D-CNN with attention module). COVNet 10 is based on a 2D CNN with ResNet-50 as a backbone to learn slice-level features which are aggregated to obtain global features. Different ResNet variants have been employed in various studies 7 8 9 11 30 . In contrast to 2D CNN, DeCoVnet 14 utilizes 3D ResBlocks and learns volumetric features using 3D convolutions. Compared to 2D CNN, it exploits multiple slices simultaneously and learns rich features. AD3D-MIL 15 additionally utilizes attention-based pooling that focuses on the most important instances for making a decision.

Using the validation sets, the three models achieved good and comparable performance. The similar mean accuracies and low standard deviations indicate the stability of the models’ performance across the folds ( Table 4 ). Using the test dataset, our classification results (obtained by an ensemble of the five best models), achieved good accuracy (Acc=73.9 – 76.6%) with high specificity (Sp=85.7 – 90.8%) and moderate sensitivity (Se=57.7 – 67.8%). The performance assessment shows that these models are useful in distinguishing between COVID-19 and nCP with good discriminating performance (AUC=0.84 – 0.86). Moreover, the models performed well on both internal (AUC=0.87, 0.88) and external (AUC=0.81 – 0.84) test sets. However, because of the only moderate sensitivity, their unsupervised clinical use is not recommended. Yet, they can potentially be used to assist radiologists. AI assistance to radiologists in differentiating between COVID-19 and nCP has been found to increase performance in terms of accuracy, diagnostic time, and diagnostic confidence 32 . In addition to the predicted classes, model confidence might play a role in certain clinical applications which will benefit from the model’s recalibration.

Other studies that evaluated COVNet and DeCoVnet with their independent datasets have reported varying levels of performance ( 11 12 13 30 ; see Table 5 ). Hou et al. 30 and Qian et al. 11 reported that COVNet yielded accuracies lower than 70% (68.2% and 68.8%, respectively) for their respective datasets. This is lower than the accuracy we obtained from COVNet (76.6%). In the present study, the evaluated models achieved similar performance, which is analogous to the previous findings of Wang et al. 12 . The authors obtained comparable sensitivities from COVNet (Se=89.8%) and DeCoVnet (Se=91.1%). In another report from Wang et al. 13 , COVNet (Acc=93.8%) performed slightly better than DeCoVnet (Acc=90.3%). However, DeCoVnet performed better than COVNet in the other studies 11 12 30 . These findings indicate that the superior performance of 2D CNN and 3D CNN depends on the underlying dataset.

Compared to the performance we achieved, first reports of COVNet 10 , DeCoVnet 14 , and AD3D-MIL 15 as well as the other studies 11 12 13 30 reported >90% performance (Acc >90% or AUC >0.95 or Se >95%) for their proposed models ( Table 5 ). One reason could be the inclusion of healthy controls in the dataset. In our present study, we focused on COVID-19 and nCP patient data with pulmonary infiltrations only. The reason being that distinguishing healthy controls from COVID-19 patients is a relatively easier task. Pneumonia in the nCP class and COVID-19 class might show similar disease patterns on CT scans 45 . Similarities in the imaging pattern make the classification problem challenging even for experienced radiologists. COVID-19 pneumonia can show a large overlap of imaging patterns with non-COVID infective lung disease, which limits the diagnostic performance of CT 46 . Our moderate classification performance is therefore in line with this commonly acknowledged limitation.

Another reason for the difference in the performance we achieved and the performance reported in the other studies could stem from dataset diversity. The studies shown in Table 5 often use a small dataset collected from local sources, i.e., data from one center 11 14 30 or from multiple centers in a single country 10 12 15 , and do not describe the inclusion of different COVID-19 stages 11 13 . In contrast, our large, diverse, and balanced dataset includes images from different scanners from China and Germany. Moreover, our data also covers different disease stages (see examples in Fig. 4 ). The COVID-19 set includes scans with early, progressive, peak, absorption stages. Viral and bacterial pneumonias in the nCP set have mild, moderate, and extensive severities. Although this diversity in the dataset may result in lower performance metrics, it is also important to consider that the AI algorithms are better equipped to address radiologically relevant cases that require accurate differentiation support.

The models trained in this study have the advantage of firstly not including healthy subjects in the training dataset, secondly having a heterogeneous disease stage within the COVID-19 cases, and thirdly requiring differentiation between viral as well as bacterial non-COVID-19 cases. As a result, the models might be more focused on the challenging cases where augmented diagnostic decision-making could offer clinical benefits.

In addition to the architectural differences, the models use slightly different preprocessing, different initialization, and in our experiments, employed different lung segmentation models. Despite these differences, the three models achieved comparable performance. As discussed above, these architectures achieved high performance using different datasets in their original publications. However, based on our diverse dataset with the two classes having similar patterns, their performance is inferior. This implies that the performance is not dependent on the architecture but rather the training data. This main finding of our study indicates that the classification performance is highly dependent on the training data rather than the underlying CNN architecture itself.

This study has certain limitations. This retrospective study design focuses on COVID-19 with exclusion of other classification options in daily clinical reporting. Moreover, human-machine interaction was not evaluated. One of the model selection criteria for this study was the ability to train with only patient-level labels. Although these labels can be easily obtained and enable quick experimentation with the AI models, diagnostic performance using slice-/pixel-level labels was not explored in this study. Follow-up studies should also evaluate AI models exploiting clinical parameters along with CT imaging. In the future, an extended dataset with scans from other centers as well as public datasets could be used. Furthermore, understanding the model’s prediction using explainable AI techniques as well as analyzing model’s confidence will also be a focus of our future studies.

## Conclusion

In summary, we trained and compared three AI models for diagnosing COVID-19. The models trained on our diverse dataset resulted in comparable performance for the independent test dataset despite fundamental algorithmic differences. The heterogeneity of the training data and the considered classification options determine the diagnostic performance. The only moderate performance of all included models with respect to the independent test set underlines that these models should not be used unsupervised but rather as a tool to assist radiologists.

## Supplementary Material

Supplementary Material Zusatzmaterial
