# Supplementary material for: Performance of AI Approaches for COVID-19 Diagnosis Using Chest CT Scans: The Impact of Architecture and Dataset
Source: Rofo. 2025 Apr 29;198(2):185–98. doi: 10.1055/a-2577-3928 (PMC12851823; doi:10.1055/a-2577-3928)
Supplement: Supplementary file 1 — Supplementary Material [file 10-1055-a-2577-3928_25780955.pdf]

Supplementary Material 1

Supplementary method:

We searched Google Scholar to find research papers published between the years 2020 and 2021, using the phrases "COVID-19 classification based on chest CT", "COVID-19 detection based on chest CT using deep learning", "artificial intelligence for COVID-19 diagnosis", etc. Subsequently, articles were screened on the basis of relevance, availability of code, quality, and the number of citations. This search was aided by (1) the search within the publications citing the studies found in the previous step, and (2) a GitHub search for repositories, using the phrases "COVID-19 classification" or "COVID-19 detection based on CT scans". Finally, we identified 15 publications that presented COVID-19 detection methods based on chest CT and shared the corresponding code. This list includes all the publications found in the systematic review by Robert et al. [1], who share their code for COVID-19 detection based on CT scans (i.e., [2–4]). Compared to [1], we found significantly (5 times) more publications (see Table 1). Note that, despite the effort to find all relevant articles with code, the goal was not to perform an exhaustive search due to the overwhelming volume of work published in this field.

Supplementary Table 1: Publications along with links to the code. Reasons for inclusion in or exclusion from our evaluation are also described.

	Publications	Code link	Included	Reason
1	Li et al. (2020) [5]	https://github.com/bkong999/COVNet	Yes	Availability of code and clear documentation. Model can only train with patient-level labels.
2	Wang et al. (2020) [6]	https://github.com/sydney0zq/covid-19-detection	Yes	Availability of code and clear documentation. Model can only train with patient-level labels
3	Han et al. (2020) [7]	https://github.com/zhyhan/AD3DMIL	Yes	Availability of code and clear documentation. Model can only train with patient-level labels
4	Jin et al. (2020) [8]	https://github.com/ChenWWWeixiang/diagnosis_covid19	No	This ResNet-151 model was excluded because ResNet-50 was already included.
5	Mei et al. (2020) [2]	https://github.com/howchihlee/COVID19_CT	No	Lack of documentation.
6	Gunraj et al. (2020) [9]	https://github.com/haydengunraj/COVIDNet-CT	No	The model only made slice-level predictions.
7	Javaheri et al. (2021) [10]	https://github.com/mohofar/CovidCtNet	No	Code could not be executed due to unresolved dependency.
8	Wang et al. (2020) [3]	https://github.com/wangshuocas/COVID-19	No	Lack of detailed documentation.
9	Xiong et al. (2020) [4]	https://github.com/robinwang08/COVID19	No	Lack of detailed documentation.
10	Rahimzadeh et al. (2021) [11]	https://github.com/mr7495/COVID-CT-Code	No	Slice-level annotations required for training.
11	Wu et al. (2021) [12]	https://github.com/yuhuan-wu/JCS	No	Pixel-level labels required for training.
12	Hou et al. (2021) [13]	https://github.com/FDU-VTS/Periphery-aware-COVID	No	Lesion annotations (pixel-level labels) required for training.
13	Lee et al. (2021) [14]	https://github.com/edhlee/Deep-COVID-DeteCT	No	Lack of detailed documentation.
14	Zhang et al. (2020) [15]	http://ncov-ai.big.ac.cn/download?lang=en	No	Pixel-level annotations required for training.
15	Song et al. (2021) [16]	https://github.com/SY575/COVID19-CT	No	Input is required in JPEG format.

Supplementary Table 2: Hyperparameters used for training the three Covid-19 detection models

	**COVNet, DeCoVnet, AD3D-MIL**
**Optimizer**	Adam
**Learning rate**	1e-5
**Epochs**	Dynamic, early stopping
**Loss function**	Cross-entropy loss

Supplementary Table 3: DeLong test for pairwise comparison of the AUCs of the three COVID-19 detection models. In addition, AUCs for external test set were compared to AUCs for the internal test set (test set from Germany vs. test set from China).

	**COVNet vs. DeCoVnet**	**COVNet vs. AD3D-MIL**	**DeCoVnet vs. AD3D-MIL**		**Test set from Germany vs. Test set from China**
**Test set from Germany**	*p* =.92	*p* =.08	*p* =.07	**COVNet**	*p* =.28
**Test set from China**	*p* =.66	*p* =.75	*p* =.94	**DeCoVnet**	*p* =.15
**Test set (Germany and China)**	*p* =.99	*p* =.17	*p* =.13	**AD3D-MIL**	*p* =**.01**

References:

[1] M. Roberts, D. Driggs, M. Thorpe, J. Gilbey, M. Yeung, S. Ursprung, A.I. Aviles-Rivero, C. Etmann, C. McCague, L. Beer, J.R. Weir-McCall, Z. Teng, E. Gkrania-Klotsas, A. Ruggiero, A. Korhonen, E. Jefferson, E. Ako, G. Langs, G. Gozaliasl, G. Yang, H. Prosch, J. Preller, J. Stanczuk, J. Tang, J. Hofmanninger, J. Babar, L.E. Sánchez, M. Thillai, P.M. Gonzalez, P. Teare, X. Zhu, M. Patel, C. Cafolla, H. Azadbakht, J. Jacob, J. Lowe, K. Zhang, K. Bradley, M. Wassin, M. Holzer, K. Ji, M.D. Ortet, T. Ai, N. Walton, P. Lio, S. Stranks, T. Shadbahr, W. Lin, Y. Zha, Z. Niu, J.H.F. Rudd, E. Sala, C.B. Schönlieb, Common pitfalls and recommendations for using machine learning to detect and prognosticate for COVID-19 using chest radiographs and CT scans, Nat Mach Intell. 3 (2021) 199–217. https://doi.org/10.1038/s42256-021-00307-0.

[2] X. Mei, H.C. Lee, K. yue Diao, M. Huang, B. Lin, C. Liu, Z. Xie, Y. Ma, P.M. Robson, M. Chung, A. Bernheim, V. Mani, C. Calcagno, K. Li, S. Li, H. Shan, J. Lv, T. Zhao, J. Xia, Q. Long, S. Steinberger, A. Jacobi, T. Deyer, M. Luksza, F. Liu, B.P. Little, Z.A. Fayad, Y. Yang, Artificial intelligence–enabled rapid diagnosis of patients with COVID-19, Nat Med. 26 (2020) 1224–1228. https://doi.org/10.1038/s41591-020-0931-3.

[3] S. Wang, Y. Zha, W. Li, Q. Wu, X. Li, M. Niu, M. Wang, X. Qiu, H. Li, H. Yu, W. Gong, Y. Bai, L. Li, Y. Zhu, L. Wang, J. Tian, A fully automatic deep learning system for COVID-19 diagnostic and prognostic analysis, European Respiratory Journal. 56 (2020). https://doi.org/10.1183/13993003.00775-2020.

[4] Z. Xiong, R. Wang, H.X. Bai, K. Halsey, J. Mei, Y.H. Li, M.K. Atalay, X.L. Jiang, F.X. Fu, L.T. Thi, R.Y. Huang, W.H. Liao, I. Pan, J.W. Choi, Q.H. Zeng, B. Hsieh, D. CuiWang, R. Sebro, P.F. Hu, K. Chang, L.B. Shi, Z.Y. Qi, Artificial Intelligence Augmentation of Radiologist Performance in Distinguishing COVID-19 from Pneumonia of Other Origin at Chest CT, Radiology. 296 (2020) E156–E165. https://doi.org/10.1148/radiol.2020201491.

[5] L. Li, L. Qin, Z. Xu, Y. Yin, X. Wang, B. Kong, J. Bai, Y. Lu, Z. Fang, Q. Song, K. Cao, D. Liu, G. Wang, Q. Xu, X. Fang, S. Zhang, J. Xia, J. Xia, Using Artificial Intelligence to Detect COVID-19 and Community-acquired Pneumonia Based on Pulmonary CT: Evaluation of the Diagnostic Accuracy, Radiology. 296 (2020) E65–E71. https://doi.org/10.1148/radiol.2020200905.

[6] X. Wang, X. Deng, Q. Fu, Q. Zhou, J. Feng, H. Ma, W. Liu, C. Zheng, A Weakly-Supervised Framework for COVID-19 Classification and Lesion Localization from Chest CT, IEEE Trans Med Imaging. 39 (2020) 2615–2625. https://doi.org/10.1109/TMI.2020.2995965.

[7] Z. Han, B. Wei, Y. Hong, T. Li, J. Cong, X. Zhu, H. Wei, W. Zhang, Accurate Screening of COVID-19 Using Attention-Based Deep 3D Multiple Instance Learning, IEEE Trans Med Imaging. 39 (2020) 2584–2594. https://doi.org/10.1109/TMI.2020.2996256.

[8] C. Jin, W. Chen, Y. Cao, Z. Xu, Z. Tan, X. Zhang, L. Deng, C. Zheng, J. Zhou, H. Shi, J. Feng, Development and evaluation of an artificial intelligence system for COVID-19 diagnosis, Nat Commun. 11 (2020). https://doi.org/10.1038/s41467-020-18685-1.

[9] H. Gunraj, L. Wang, A. Wong, COVIDNet-CT: A Tailored Deep Convolutional Neural Network Design for Detection of COVID-19 Cases From Chest CT Images, Front Med (Lausanne). 7 (2020). https://doi.org/10.3389/fmed.2020.608525.

[10] T. Javaheri, M. Homayounfar, Z. Amoozgar, R. Reiazi, F. Homayounieh, E. Abbas, A. Laali, A.R. Radmard, M.H. Gharib, S.A.J. Mousavi, O. Ghaemi, R. Babaei, H.K. Mobin, M. Hosseinzadeh, R. Jahanban-Esfahlan, K. Seidi, M.K. Kalra, G. Zhang, L.T. Chitkushev, B. Haibe-Kains, R. Malekzadeh, R. Rawassizadeh, CovidCTNet: an open-source deep learning approach to diagnose covid-19 using small cohort of CT images, NPJ Digit Med. 4 (2021). https://doi.org/10.1038/s41746-021-00399-3.

[11] M. Rahimzadeh, A. Attar, S.M. Sakhaei, A fully automated deep learning-based network for detecting COVID-19 from a new and large lung CT scan dataset, Biomed Signal Process Control. 68 (2021). https://doi.org/10.1016/j.bspc.2021.102588.

[12] Y.H. Wu, S.H. Gao, J. Mei, J. Xu, D.P. Fan, R.G. Zhang, M.M. Cheng, JCS: An Explainable COVID-19 Diagnosis System by Joint Classification and Segmentation, IEEE Transactions on Image Processing. 30 (2021) 3113–3126. https://doi.org/10.1109/TIP.2021.3058783.

[13] J. Hou, J. Xu, L. Jiang, S. Du, R. Feng, Y. Zhang, F. Shan, X. Xue, Periphery-aware COVID-19 diagnosis with contrastive representation enhancement, Pattern Recognit. 118 (2021). https://doi.org/10.1016/j.patcog.2021.108005.

[14] E.H. Lee, J. Zheng, E. Colak, M. Mohammadzadeh, G. Houshmand, N. Bevins, F. Kitamura, E. Altinmakas, E.P. Reis, J.K. Kim, C. Klochko, M. Han, S. Moradian, A. Mohammadzadeh, H. Sharifian, H. Hashemi, K. Firouznia, H. Ghanaati, M. Gity, H. Doğan, H. Salehinejad, H. Alves, J. Seekins, N. Abdala, Ç. Atasoy, H. Pouraliakbar, M. Maleki, S.S. Wong, K.W. Yeom, Deep COVID DeteCT: an international experience on COVID-19 lung detection and prognosis using chest CT, NPJ Digit Med. 4 (2021). https://doi.org/10.1038/s41746-020-00369-1.

[15] K. Zhang, X. Liu, J. Shen, Z. Li, Y. Sang, X. Wu, Y. Zha, W. Liang, C. Wang, K. Wang, L. Ye, M. Gao, Z. Zhou, L. Li, J. Wang, Z. Yang, H. Cai, J. Xu, L. Yang, W. Cai, W. Xu, S. Wu, W. Zhang, S. Jiang, L. Zheng, X. Zhang, L. Wang, L. Lu, J. Li, H. Yin, W. Wang, O. Li, C. Zhang, L. Liang, T. Wu, R. Deng, K. Wei, Y. Zhou, T. Chen, J.Y.N. Lau, M. Fok, J. He, T. Lin, W. Li, G. Wang, Clinically Applicable AI System for Accurate Diagnosis, Quantitative Measurements, and Prognosis of COVID-19 Pneumonia Using Computed Tomography, Cell. 181 (2020) 1423-1433.e11. https://doi.org/10.1016/j.cell.2020.04.045.

[16] Y. Song, S. Zheng, L. Li, X. Zhang, X. Zhang, Z. Huang, J. Chen, R. Wang, H. Zhao, Y. Chong, J. Shen, Y. Zha, Y. Yang, Deep Learning Enables Accurate Diagnosis of Novel Coronavirus (COVID-19) with CT Images, IEEE/ACM Trans Comput Biol Bioinform. 18 (2021) 2775–2780. https://doi.org/10.1109/TCBB.2021.3065361.
